# Active Surveillance of Hansen's Disease (Leprosy): Importance for Case Finding among Extra-domiciliary Contacts

**DOI:** 10.1371/journal.pntd.0002093

**Published:** 2013-03-14

**Authors:** Maria L. N. Moura, Kathryn M. Dupnik, Gabriel A. A. Sampaio, Priscilla F. C. Nóbrega, Ana K. Jeronimo, Jose M. do Nascimento-Filho, Roberta L. Miranda Dantas, Jose W. Queiroz, James D. Barbosa, Gutemberg Dias, Selma M. B. Jeronimo, Marcia C. F. Souza, Maurício L. Nobre

**Affiliations:** 1 Department of Biochemistry, Biosciences Center, Federal University of Rio Grande do Norte, Natal, RN, Brazil; 2 Division of Infectious Diseases, Weill Cornell Medical College, New York, New York, United States of America; 3 Institute of Science and Technology of Tropical Diseases (INCT-DT), Natal, RN, Brazil; 4 Health Post-Graduate Program, Health Sciences Center, Federal University of Rio Grande do Norte, Natal, RN, Brazil; 5 State University of Rio Grande do Norte, Mossoró, RN, Brazil; 6 National Institute of Social Security (INSS), Mossoró, RN, Brazil; 7 Post-Graduate Graduate Program in Tropical Medicine, Oswaldo Cruz Institute, Fiocruz, Rio de Janeiro, RJ, Brazil; 8 Hospital Giselda Trigueiro, Natal, RN, Brazil; Fondation Raoul Follereau, France

## Abstract

Hansen's disease (leprosy) remains an important health problem in Brazil, where 34,894 new cases were diagnosed in 2010, corresponding to 15.3% of the world's new cases detected in that year. The purpose of this study was to use home visits as a tool for surveillance of Hansen's disease in a hyperendemic area in Brazil. A total of 258 residences were visited with 719 individuals examined. Of these, 82 individuals had had a previous history of Hansen's disease, 209 were their household contacts and 428 lived in neighboring residences. Fifteen new Hansen's disease cases were confirmed, yielding a detection rate of 2.0% of people examined. There was no difference in the detection rate between household and neighbor contacts (p = 0.615). The two groups had the same background in relation to education (p = 0.510), household income (p = 0.582), and the number of people living in the residence (p = 0.188). Spatial analysis showed clustering of newly diagnosed cases and association with residential coordinates of previously diagnosed multibacillary cases. Active case finding is an important tool for Hansen's disease control in hyperendemic areas, enabling earlier diagnosis, treatment, decrease in disability from Hansen's disease and potentially less spread of *Mycobacterium leprae*.

## Author Summary

Hansen's Disease, or leprosy, is a disease that despite curative therapy is still a health problem in many areas, particularly in Brazil, which has a high new case detection rate. If symptoms of Hansen's disease are not recognized, delay in diagnosis can result in severe disability. Within the state of Rio Grande do Norte, Brazil, a state that has had a low detection rate, we focused on a municipality which is considered hyperendemic. We visited households of previously diagnosed Hansen's disease cases and two neighboring households. There was no difference in the rate of detection of new cases within case and neighbor households, nor differences with respect to education, household income, or the number of people living in the residence. By mapping these households, we found that proximity to a multibacillary case increased the risk of finding a new case of Hansen's disease. Spatial analysis in areas with Hansen's disease should be a tool for implementation of active surveillance to help reduce disease transmission. In addition, it is essential to raise awareness in communities at highest risk to promote early detection and treatment of new cases.

## Introduction

Hansen's disease, as leprosy is called in Brazil, is an infectious disease of insidious onset, caused by *Mycobacterium leprae*. [1]–[3] Transmission is thought to occur primarily via the airborne route from people with multibacillary disease. A great challenge to disease control is the identification of people at risk of infection and development of disease. [4]–[6] Time between infection and disease development can vary and be five or more years after exposure; this makes interruption of transmission more challenging and it is difficult to identify areas at highest risk. [7]–[9] In endemic areas, the majority of individuals infected with *M. leprae* do not develop disease, [10]; [11] and it is believed that disease development is associated with close and prolonged contact with untreated people with multibacillary disease, [12]; [13] as well as genetic [14]–[16] and socioeconomic factors. [17]; [18]

A significant challenge to interruption of transmission of *M. leprae* by early diagnosis of Hansen's disease is that initial skin lesions can be very discrete and asymptomatic. For this reason, different strategies for case finding have been investigated. Van Beers et al (1999) observed that the risk for Hansen's disease in a highly endemic area was higher in household contacts or neighbors with direct contact with a case, compared to households without direct contact. [19] Studies of spatial clustering have shown that physical distance can define risk groups associated with disease occurrence. Hoeven et al (2008) identified an area with radius of 10 meters from the index case as being the highest risk for development of Hansen's disease. [20]

The introduction of multidrug therapy (MDT) in 1981 resulted in a drastic shift in the global distribution of Hansen's disease, and has been responsible for a significant decrease in new case detection in the past few decades. [21], [22] Despite this advance, Hansen's disease continues to be endemic in many countries, including Brazil, which has the second highest detection rate worldwide, [23] 1.54 cases/10,000 inhabitants. [24]; [25] Rio Grande do Norte (RN), a state located in the northeast of Brazil, has traditionally had a lower case detection rate than neighboring states, yet an increase in new case detection during the last decade has been documented. [26]

The examination of household contacts of known cases has been used as a tool to increase the early diagnosis of the disease and to interrupt transmission, [27]; [28] but the utility of examination of other groups, such as neighborhood and social contacts, is less clear. Brazil's public health service is based on health teams composed of at least one doctor, one nurse, one auxiliary nurse and five paramedical workers who are responsible for 200 families in a small geographic area. Health team activities include home visits and monitoring of diseases prevalent in their area. The current study's objective was to evaluate clustering/mapping as a tool for identification of high-risk areas of Hansen's disease and the utility of skin and neurological examination during household visits in high-prevalence neighborhoods for identifying new cases of Hansen's disease.

## Methods

### 1. Study area and population

This study was conducted between January 20 and February 18, 2006 in the municipality of Mossoró, Rio Grande do Norte, Brazil, which had a population of 229,784 inhabitants in 2006 according to estimates of the Brazilian Institute for Geography and Statistics (IBGE). A database with information about known Hansen's disease cases was obtained from the Municipal Health Office and used for spatial analysis of 808 cases of the disease in the municipality as shown previously. [29] Previous active case finding in Mossoró was related to educational campaigns rather than by surveys or home visits

Two neighborhoods with the highest concentration of Hansen's disease cases in the municipality (427 cases) were selected for this work. Most of these cases had sought diagnosis at outpatient clinics. Within this group, 82 individuals with prior diagnosis of Hansen's disease (cases) agreed to take part in this study. If the case entered the study, the two neighboring households were also invited to participate. Therefore, the study population consisted of people who were previously diagnosed with Hansen's disease, their household contacts, and residents of the neighboring houses. People residing in the neighboring houses were considered to be extra-domiciliary contacts, if they hadn't had a known case of Hansen's disease in that residence. If a neighbor had a history of Hansen's disease in his or her household, this neighbor's household was considered to be a case family and the next household was invited to participate in the study. The major outcome for the study population was presence of new case of Hansen's disease among people who were either household or neighbor contacts of a previous case. Our hypothesis was that household contacts of index cases would be more likely to be diagnosed with Hansen's disease than non-household contacts.

### 2. Home visits

A team of four physicians, six medical students, one social worker, and one nurse conducted the home visits for families of previously diagnosed cases (“household contacts”) and two neighboring consenting homes. Every residence visited had its GPS coordinate determined with Teletype GPS (TCF 1358) on Pocket PC (Hewlett Packard Jornada). The program ArcMap 9.1 was used to create maps of the georeferenced residences.

### 3. Procedures

Volunteers responded to a verbally administered questionnaire on age, profession, household income, schooling, residential history, and personal or family history of diabetes, hypertension, tuberculosis, allergies, and Hansen's disease. Each person received a dermato-neurologic exam. Skin lesions suspicious for Hansen's disease were tested for light touch sensation using Semmes-Weinstein monofilaments. Persons with lesions suspicious for Hansen's disease were referred to Mossoró's health post for evaluation by a specialist physician to obtain skin smears to assess for *M. leprae* and to determine need for skin biopsy, in addition to evaluating other causes of hypopigmented skin lesions, including fungal infections.

If Hansen's disease diagnosis was confirmed, the health post physician determined degree of disability and initiated multi-drug therapy. New cases were classified according to the criteria of Ridley and Jopling. [30]; [31]

### 4. Statistical analysis

Data were stored in Microsoft Excel XP and analyzed with STATISTICA (release 6.1, StatSoft, USA). Family income was considered as the number of minimum wages earned by the household. Monthly minimum wage in Brazil in 2006 was approximately U$ 250. To analyze education level and household population density (the number of individuals per meter squared) and to compare the mean age among groups, the two-sided t-test was used.

The locations of the Hansen's disease cases diagnosed in the current study were analyzed considering their distance to the previously mapped households of 427 Hansen's disease cases diagnosed between 1995 and 2006, of whom 229 (53.6%) were multibacillary cases. Since the location of this study fell within a previously described high cluster of Hansen's disease, [29] we took into consideration three groups as events: new cases, previously diagnosed multibacillary cases, and previously diagnosed paucibacillary cases. To test the hypothesis that the distribution pattern of the newly diagnosed Hansen's disease cases was independent of previous cases (either multibacillary or paucibacillary), Monte Carlo simulations were performed with nsim = 39 replication. The analysis estimated the G_cross_ function, G_ij_(r), for each pair of groups comparing new Hansen's disease cases to Hansen's disease cases diagnosed previously (either multibacillary or paucibabacillary cases), composing multi-type processes. The G_cross_ function G_ij_(r) estimates the probability that the distance from a point in the i group to the nearest point in the j group falls into a circle of ray r. The probability is then represented in the Y axis of the graph. The theoretical distribution of the distances under independence hypotheses between the groups i and j, where the j group has intensity 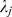, has the form 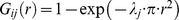 Deviations between the empirical and theoretical Gij curves may suggest dependence between the points of types i and j. An envelope with one sided p-value of p = 1/(nsim+1) = 2.5%, yielded a 95% confidence interval for each pair of *G_ij_* curves. Dependence may be suggested when at least part of a *G_ij_* curve is found above the high limit of its interval. The spatstat package in R (version 2.12.1 http://www.r-project.org) was used to perform the analysis.

### 5. Ethical considerations

All individuals were educated regarding the objectives of the study using an informed consent form. The consent form and study protocol were approved by the Research Ethics Committee of the Federal University of Rio Grande do Norte as well as by the National Research Ethics Committee (CEP-UFRN 145/05; CONEP 12504, CAAE 006.0.051000-06).

## Results

### 1. Home visits as a tool for new case diagnosis in a hyperendemic area

A total of 258 residences were visited and 719 people were examined. Table 1 shows the ages of people examined. Of the studied subjects, 82 were previous cases of Hansen's disease, 209 were household contacts and 428 were neighbors. Of the 202 families with a history of Hansen's disease, 41 (20.3%) had more than one case of Hansen's disease in the family (mean 3.8 cases, with range from 2 to 8 Hansen's disease cases per family) (Table S1).

**Table 1:** The age distribution of individuals examined in the present study.

Age group (years)	Individuals			Total n (%)
	Previous Hansen's disease case	Household contact	Neighbor contact	
	n (%)	n (%)	n (%)	
0–10	1 (1.2)	39 (18. 7)	70 (16.4)	110 (15.3)
11–20	5 (6.1)	47 (22.5)	102 (23.3)	154 (21.4)
21–30	13 (15.9)	36 (17.2)	65 (15.2)	114 (15.9)
31–40	12 (14.6)	28 (13.4)	58 (13.5)	98 (13.6)
41–50	15 (18.3)	22 (10. 5)	50 (11.7)	87 (12.1)
>51	36 (43.9)	37 (17.7)	83 (19.4)	156 (21.7)
Total	82 (100.0)	209 (100.0)	428 (100.0)	719 (100.0)

Based on dermatologic and neurologic examinations, there were 62 suspected Hansen's disease cases out of 637 people without a history of Hansen's disease. Clinical and histopathological examinations by a specialist confirmed the diagnosis of Hansen's disease for 15 people, which corresponded to a detection rate of 2.4 cases per 100 examinations of household and neighbor contacts (Table 2). Of these new Hansen's disease cases, 6 (40.0%) were household contacts and 9 (60.0%) were neighbor contacts, with no difference in the rate of new cases found in household (2.9/100) or neighbor (2.1/100) contacts (p = 0.555) (Table 2). Over half of study participants had household income of two or fewer minimum wages (Table S2), with no significant difference between case and neighbor households (p = 0.582). In this study population, residents had few years of schooling, but there was no difference between Hansen's disease case and neighbor household contacts (p = 0.582). Within the overall study population, 81.4% had resided in the neighborhood for four or more years (Table 3).

**Table 2:** Household type (household versus neighbor) of newly detected leprosy cases.

Newly diagnosed Hansen's case	Type of contact		Total n (%)
	Household n (%)	Neighbor n (%)	
yes	6 (2.9)	9 (2.1)	15 (2.4)
no	203 (97.1)	419 (97.9)	622 (97.6)
Total	209 (100.0)	428 (100.0)	637 (100.0)

p = 0.555.

**Table 3:** Years living in the neighborhood.

Years living in the area	Number of subjects	%
<1	55	7.6
2	28	3.9
3	49	6.8
4	77	10.8
≥5	509	70.8
Not known	1	0.1
Total	719	100.0

The mean age of previously diagnosed Hansen's disease cases (46.4± SD 18.5 years) was significantly higher than household contacts (30.3±21.2 years) (p<0.0001) and neighbor contacts (31.5±21.3 years) (p<0.0001). No difference in age (p = 0.5221) or gender (p = 0.881) between household contacts and neighbor contacts was observed. Newly diagnosed Hansen's disease cases were younger than previously diagnosed Hansen's disease cases, 34.4 (±17.7) years vs. 46.4 (±18.5) years, respectively (p = 0.0220). Of the new cases, four (26.7%) were less than 20 years old and 8 (53.3%) were males. The clinical classification of the cases was confirmed with histopathology of skin biopsies using the criteria of Ridley and Jopling (Table 4). After confirmation of diagnosis, new cases were started on multidrug therapy as recommended by the World Health Organization.(18) Of the 15 new cases, ten had WHO disability grade zero, three had disability grade 1, and two had disability grade 2.

**Table 4:** Clinical classification of new Hansen's disease cases diagnosed during the study.

Clinical presentation	Number of cases	%
TT	4	26.6
BT	3	20.0
BB	1	6.7
BL	2	13.3
LL	1	6.7
Indeterminate	4	26.7
Total	15	100

### 2. Spatial analysis of newly diagnosed cases of Hansen's disease

**Figure 1:**
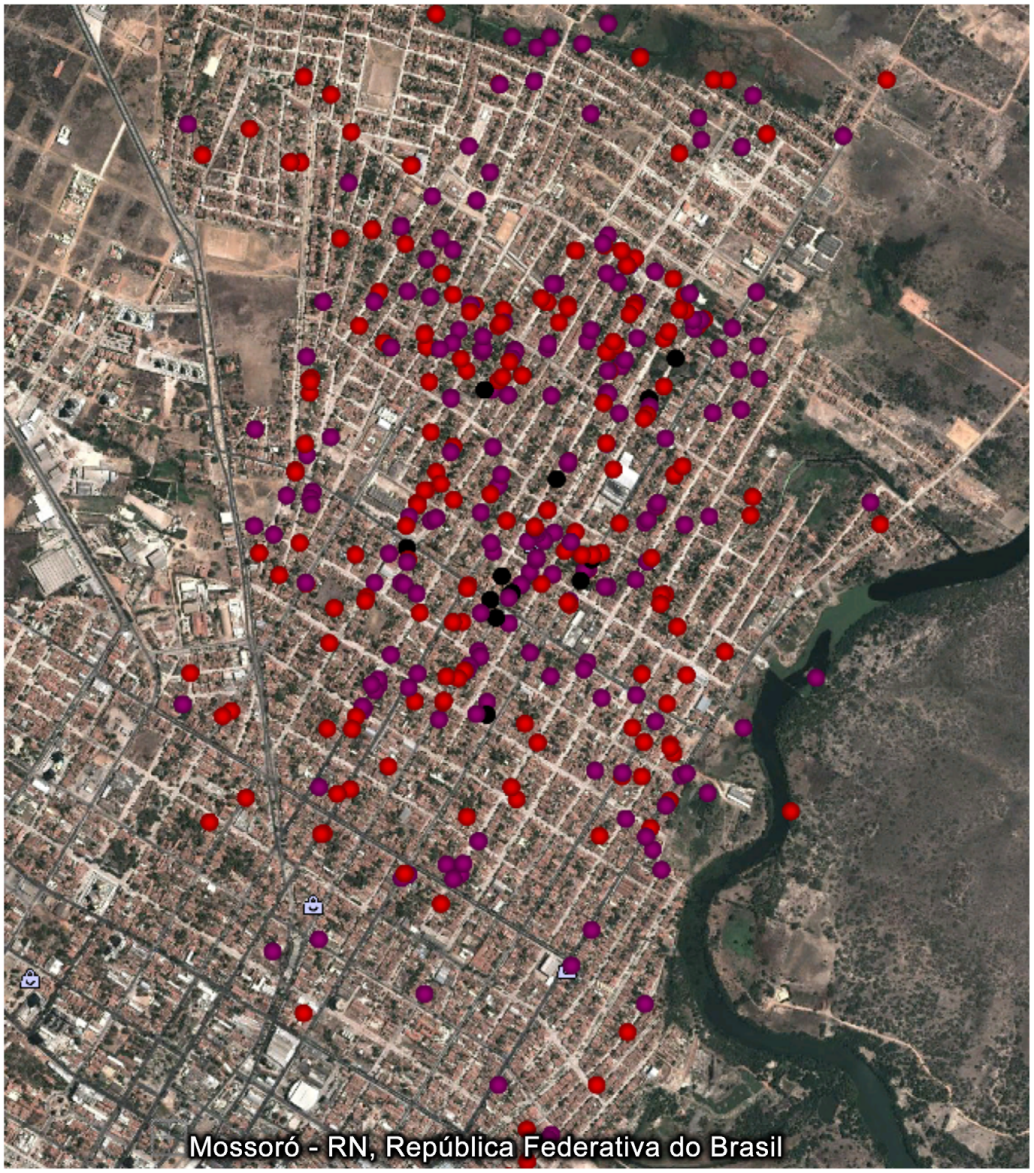
Spatial distribution of new (black) and previous paucibacillary (purple) and multibacillary (red) cases.

**Figure 2:**
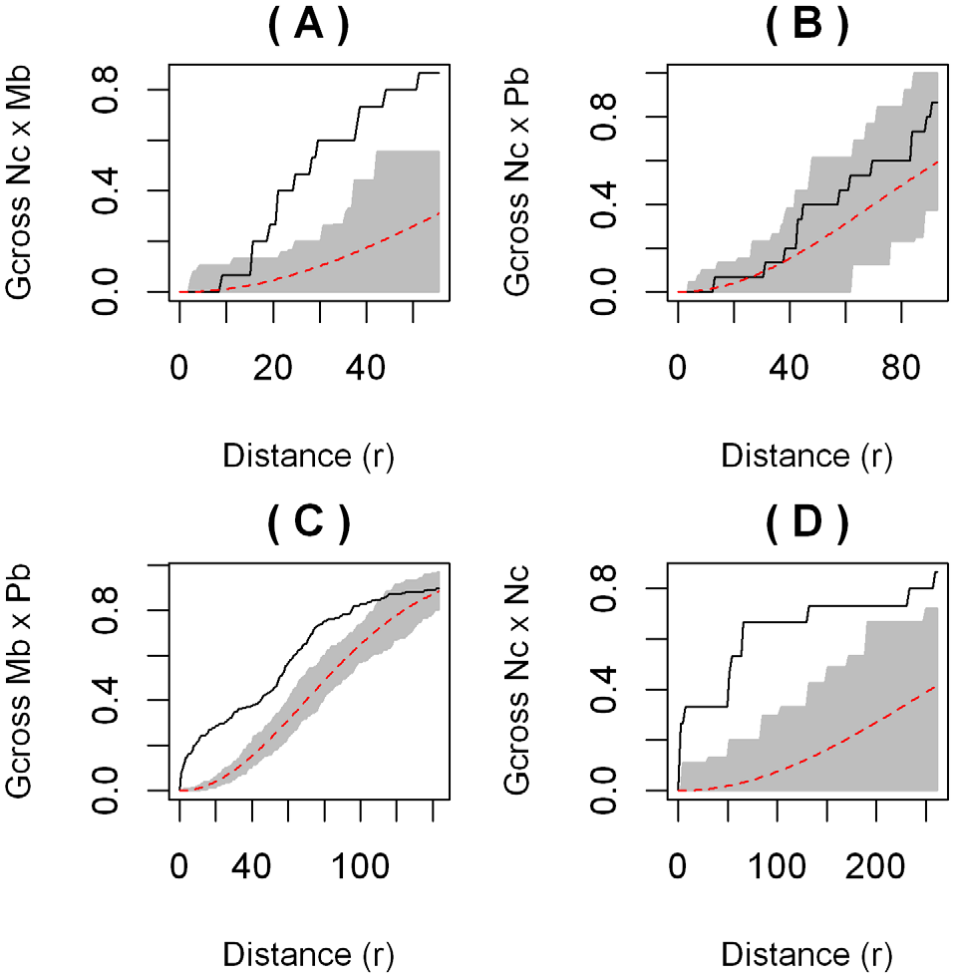
Gcross analysis of Hansen's disease. A. New (Nc) versus previous multibacillary cases (Mb). B. New (Nc) versus previous paucibacillary cases (Pb). C. Previous Multibacillary (Mb) versus Paucibacillary cases (Pb). D. New Hansen's disease cases (Nc).

The geographic distribution of the newly diagnosed Hansen's disease cases (n = 15) with respect to 427 previous Hansen's disease cases (clustered area), of which 229 (53.6%) were multibacillary cases is shown in Figure 1. The hypothesis that the new case household locations were independent from the previous multibacillary cases' households was rejected, as shown in Figure 2A, since the observed Gcross curve is found above the theoretical curve. The hypothesis was not rejected when paucibacillary cases were considered (Figure 2B). Furthermore, the distribution of paucibacillary cases was dependent on presence of multibacillary cases (Figure 2C). The newly diagnosed Hansen's disease case distribution was not random; rather it was clustered, as shown in Figure 2D, and was dependent on the presence of multibacillary cases (Figure 2A).

## Discussion

Hansen's disease remains an important public health problem in many areas of the world and Brazil contributes the second highest number of new cases worldwide after India. Although curative therapy has resulted in a substantial decrease in the number of cases, there is still a need for better strategies for disease control and prevention of disability in affected individuals. Active case finding is used in some areas as a tool for attainment of these objectives as it permits earlier diagnosis of cases in the community with decrease in degree of disability at diagnosis and interruption of transmission. Studies of spatial clustering show that physical distance can define risk groups associated with disease occurrence. [20], [32], [33] In this study, the difference in detection rates between household contacts (2.9/100) and neighbors (2.1/100) was not significant. Such results demonstrate the importance of expanding the scope of contact investigations to include residents in neighboring homes, particularly in hyperendemic areas with a high population density where risk may be elevated community-wide rather than just in the households of cases.

Our results agree with other studies which showed that in hyperendemic areas the risk of disease is high in social contacts. [20]; [34], [35] The mean age of the previously diagnosed cases was older than the contacts, similar to findings of Moet et al who showed that age was an independent risk factor for developing the disease. [34] However, the newer cases were younger, with four (27%) less than 20 years old, which suggests an early exposure to *M. leprae* in this hyperendemic area. This is an important finding which suggests that passive case detection may result in later diagnoses. The newly diagnosed cases were of the same mean age as the household and neighbor contacts without Hansen's disease.

The association of Hansen's disease with areas of high population density and poverty has been reported in the literature, [25]; [36]–[38] and we found no differences in these parameters between cases, household contacts, or neighbor contacts. However, there was a difference in relation to other regions in the municipality; the study participants lived in neighborhoods of worse socioeconomic status as determined by household income, population density and education. Queiroz et al, 2010, analyzing the overall case distribution of Hansen's disease in this municipality, found that the risk of disease was associated with factors related to poverty, although a model including measures of poverty could not explain entirely the clustering observed. [29]

In this study, we saw clusters of Hansen's disease in family groups with up to eight cases in a single family; this type of clustering has also been reported in Indonesia. [39] A study by Deps et al. in Brazil showed that a large number of patients diagnosed with Hansen's disease had a member of their family with the disease. [40] In addition, numerous studies including genome-wide association studies have suggested a genetic component to the risk of developing Hansen's disease. [15], [41], [42] Clinical investigation of all household contacts of newly diagnosed cases is recommended by the Brazilian Ministry of Health as an important tool for new case detection (http://portal.saude.gov.br/portal/arquivos/pdf/portaria_n_3125_hanseniase_2010.pdf), but this investigation is usually done at health posts and not during home visits. Our study shows the importance of including neighborhood contacts in skin and neurologic examinations for Hansen's disease, especially those who live close to a multibacillary case. Therefore, a greater involvement of health teams in home-based diagnosis and surveillance is important in areas with high risk of exposure. The structure of the public health system in Brazil, especially its team-based community health strategy, can significantly contribute to Hansen's disease control if home visits are routinely used as an opportunity to screen members of hyperendemic communities.

## Supporting Information

Checklist S1 Strobe checklist.
(DOC)

Table S1 Number of known Hansen's disease cases per family.
(DOC)

Table S2 Household income of the study population.
(DOC)

## References

[1] Sharma VK (1968) The epidemiologic significance of leprosy within the household. Int J Lepr Other Mycobact Dis 36: 1–16. 5689647

[2] Bechelli LM (1973) Advances in leprosy control in the last 100 years. Int J Lepr Other Mycobact Dis 41: 285–97. 4591732

[3] Britton WJ, Lockwood DN (2004) Leprosy. Lancet 363: 1209–19. 15081655 10.1016/S0140-6736(04)15952-7

[4] Jesudasan K, Bradley D, Smith PG, Christian M (1984) The effect of intervals between surveys on the estimation of incidence rates of leprosy. Lepr Rev 55: 353–9. 6335544 10.5935/0305-7518.19840040

[5] Shields ED, Russell DA, Pericak-Vance MA (1987) Genetic epidemiology of the susceptibility to leprosy. J Clin Invest 79: 1139–43. 3549780 10.1172/JCI112930 PMC424295

[6] Kai M, Maeda Y, Maeda S, Fukutomi Y, Kobayashi K, Kashiwabara Y, et al (2004) Active surveillance of leprosy contacts in country with low prevalence rate. Int J Lepr Other Mycobact Dis 72: 50–3. 15217314 10.1489/1544-581X(2004)072<0050:ASOLCI>2.0.CO;2

[7] Prasad KV, Ali PM (1967) Incubation period of leprosy. Indian J Med Res 55: 29–42. 6036049

[8] Pearce V, Horton JJ (2008) Leprosy: recognizing red flags. Australas J Dermatol 49: 226–8. 18855787 10.1111/j.1440-0960.2008.00475.x

[9] Suzuki K, Udono T, Fujisawa M, Tanigawa K, Idani G, et al (2010) Infection during infancy and long incubation period of leprosy suggested in a case of a chimpanzee used for medical research. J Clin Microbiol 2010 48: 3432–4. 10.1128/JCM.00017-10 PMC2937736 20631101

[10] Godal T, Negassi K (1973) Subclinical infection in leprosy. Br Med J 3: 557–9. 4580217 10.1136/bmj.3.5880.557 PMC1586807

[11] Goulart LR, Goulart IM (2009) Leprosy pathogenetic background: a review and lessons from other mycobacterial diseases. Arch Dermatol Res 2009 301: 123–37. 10.1007/s00403-008-0917-3 19043725

[12] Doull JA, Guinto RS (1947) Historical inquiry as a method of estimating the trend of leprosy. Int J Lepr 15: 369–77. 18919923

[13] Guinto RS, Doull JA, Bancroft H, Rodriguez JN (1951) A field study of leprosy in Cordova, Philippines; resurvey in 1941 after eight years. Int J Lepr 19: 117–35. 14873379

[14] Liu H, Irwanto A, Tian H, Fu X, Yu Y, et al (2012) Identification of IL18RAP/IL18R1 and IL12B as leprosy risk genes demonstrates shared pathogenesis between inflammation and infectious diseases. Am J Hum Genet 91: 935–41. 23103228 10.1016/j.ajhg.2012.09.010 PMC3487119

[15] Netea MG, Kullberg BJ, van der Meer JW (2010) Genomewide association study of leprosy. N Engl J Med 362: 1447–8. 20397292

[16] Wheeler E, Miller EN, Peacock CS, Donaldson IJ, Shaw MA, et al (2006) Genome-wide scan for loci influencing quantitative immune response traits in the Belem family study: comparison of methods and summary of results. Ann Hum Genet 70 (Pt 1) 78–97. 16441259 10.1111/j.1529-8817.2005.00223.x

[17] van Veen NH, McNamee P, Richardus JH, Smith WC (2009) Cost-effectiveness of interventions to prevent disability in leprosy: a systematic review. PLoS One 4: e4548. 19229328 10.1371/journal.pone.0004548 PMC2639641

[18] Hotez PJ, Ferris MT (2006) The antipoverty vaccines. Vaccine 24 (31–32) 5787–99. 16759763 10.1016/j.vaccine.2006.05.008

[19] Van Beers SM, Hatta M, Klatser PR (1999) Patient contact is the major determinant in incident leprosy: implications for future control. Int J Lepr Other Mycobact Dis 67: 119–28. 10472363

[20] Hoeven TA, Fischer EA, Pahan D, Richardus JH (2008) Social distance and spatial distance are not the same, observations on the use of GIS in leprosy epidemiology. Epidemiol Infect 136: 1624–7. 18272012 10.1017/S0950268808000381 PMC2870776

[21] Lockwood DN (2000) Leprosy in the new millennium. J Med Microbiol 49: 301–3. 10755622 10.1099/0022-1317-49-4-301

[22] Katoch K, Katoch VM, Natarajan M, Gupta UD, et al (2008) Long term follow-up results of 1 year MDT in MB leprosy patients treated with standard MDT+once a month Minocycline and Ofloxacin. Indian J Lepr 80: 331–44. 20329382

[23] WHO (2010) Global leprosy situation. Weekly epidemiological Record 85: 337–348.20830851

[24] Penna ML, de Oliveira ML, Penna GO (2009) The epidemiological behaviour of leprosy in Brazil. Lepr Rev 80: 332–44. 19961107

[25] Lindoso JA, Lindoso AA (2009) Neglected tropical diseases in Brazil. Rev Inst Med Trop Sao Paulo 51: 247–53. 19893976 10.1590/s0036-46652009000500003

[26] De Souza Dias MC, Dias GH, Nobre ML (2007) The use of Geographical Information System (GIS) to improve active leprosy case finding campaigns in the municipality of Mossoro, Rio Grande do Norte State, Brazil. Lepr Rev 78: 261–9. 18035777

[27] Shen J, Wang Y, Zhou M, Li W (2009) Analysis on value of household contact survey in case detection of leprosy at a low endemic situation in China. Indian J Dermatol Venereol Leprol 75: 152–6. 19293502 10.4103/0378-6323.48660

[28] Shen JP, Gupte MD, Jiang C, Manickam P, et al (2005) Trends of case detection and other indicators of leprosy in China during 1985–2002. Chin Med Sci J 20: 77–82. 16075742

[29] Queiroz JW, Dias GH, Nobre ML, De Sousa Dias MC, Araújo SF, et al (2010) Geographic information systems and applied spatial statistics are efficient tools to study Hansen's disease (leprosy) and to determine areas of greater risk of disease. Am J Trop Med Hyg 82: 306–14. 20134009 10.4269/ajtmh.2010.08-0675 PMC2813173

[30] Ridley DS, Jopling WH (1962) A classification of leprosy for research purposes. Lepr Rev 133: 119–28. 10.5935/0305-7518.19620014 14492126

[31] Features of the Ridley-Jopling classification (1979) Int J Lepr Other Mycobact Dis 47: 611–2. 122635

[32] Richardus JH, Meima A, van Marrewijk CJ, Croft RP, Smith TC (2005) Close contacts with leprosy in newly diagnosed leprosy patients in a high and low endemic area: comparison between Bangladesh and Thailand. Int J Lepr Other Mycobact Dis 3: 249–57. 16830634

[33] Bakker MI, Scheelbeek PF, Van Beers SM (2009) The use of GIS in leprosy control. Lepr Rev 80: 327–31. 19961106

[34] Moet FJ, Pahan D, Schuring RP, Oskam L, Richardus JH (2006) Physical distance, genetic relationship, age, and leprosy classification are independent risk factors for leprosy in contacts of patients with leprosy. J Infect Dis 193: 346–53. 16388481 10.1086/499278

[35] Richardus JH, Habbema JD (2007) The impact of leprosy control on the transmission of M. leprae: is elimination being attained? Lepr Rev 78: 330–7. 18309706

[36] Bakker MI, Hatta M, Kwenang A, Van MP, Faber WR, et al (2006) Risk factors for developing leprosy–a population-based cohort study in Indonesia. Lepr Rev 77: 48–61. 16715690

[37] Hotez PJ, Bottazzi ME, Franco-Paredes C, Ault SK, Periago MR (2008) The neglected tropical diseases of Latin America and the Caribbean: a review of disease burden and distribution and a roadmap for control and elimination. PLoS Negl Trop Dis 2: e300. 18820747 10.1371/journal.pntd.0000300 PMC2553488

[38] Murthy PK (2004) Clinical manifestations, diagnosis and classification of leprosy. J Indian Med Assoc 102: 678–9. 15871350

[39] Bakker MI, May L, Hatta M, Kwenang A, Klatser PR, et al (2005) Genetic, household and spatial clustering of leprosy on an island in Indonesia: a population-based study. BMC Med Genet 6: 40. 16307680 10.1186/1471-2350-6-40 PMC1318483

[40] Deps PD, Guedes BV, Bucker FJ, Andreatta MK, et al (2006) Characteristics of known leprosy contact in a high endemic area in Brazil. Lepr Rev 77: 34–40. 16715688

[41] Blackwell JM (2001) Modern genetics and leprosy susceptibility. Lepr Rev 72: 352–6. 11715282 10.5935/0305-7518.20010043

[42] Mira MT, Alcaïs A, Nguyen VT, Moraes MO, Di Flumeri C, Vu HT, et al (2004) Susceptibility to leprosy is associated with PARK2 and PACRG. Nature 427: 636–40. 14737177 10.1038/nature02326

