# Supplementary material for: Active Surveillance of Hansen's Disease (Leprosy): Importance for Case Finding among Extra-domiciliary Contacts
Source: PLoS Negl Trop Dis. 2013 Mar 14;7(3):e2093. doi: 10.1371/journal.pntd.0002093 (PMC3597486; doi:10.1371/journal.pntd.0002093)
Supplement: Table S1 — Number of known Hansen's disease cases per family. (DOC) [file pntd.0002093.s002.doc]

Table S1. Number of known Hansen’s disease cases per family

| Hansen’s disease cases (n) | Number of families | % |
| --- | --- | --- |
| 0 | 105 | 52.0 |
| 1 | 56 | 27.7 |
| 2 | 21 | 10.3 |
| 3 | 6 | 3.0 |
| 4 | 2 | 1.0 |
| 5 | 4 | 2.0 |
| 6 | 2 | 1.0 |
| 8 | 1 | 0.5 |
| Not known | 5 | 2.5 |
| Total | 202 | 100.0 |
