# Supplementary material for: Active Surveillance of Hansen's Disease (Leprosy): Importance for Case Finding among Extra-domiciliary Contacts
Source: PLoS Negl Trop Dis. 2013 Mar 14;7(3):e2093. doi: 10.1371/journal.pntd.0002093 (PMC3597486; doi:10.1371/journal.pntd.0002093)
Supplement: Table S2 — Household income of the study population. (DOC) [file pntd.0002093.s003.doc]

Table S2. Family income of the study population

| Income (Number of monthly minimum wage) | Neighbor family | | Case family | | Total | |
| --- | --- | --- | --- | --- | --- | --- |
|  | n | % | n | % | n | % |
| No income | 1 | 0.7 | 0 | 0.0 | 1 | 0.4 |
| 1 | 30 | 21.0 | 10 | 12.5 | 40 | 17.9 |
| >1-2 | 51 | 35.7 | 30 | 37.5 | 81 | 36.3 |
| >2-3 | 31 | 21.7 | 20 | 25.0 | 51 | 22.9 |
| >3-4 | 15 | 10.5 | 11 | 13.8 | 26 | 11.7 |
| >4 | 12 | 8.4 | 9 | 11.3 | 21 | 9.4 |
| No information | 3 | 2.1 | 0 | 0.0 | 3 | 1.3 |
| Total | 143 | 100.0 | 80 | 100.0 | 223 | 100.0 |

p = 0.582, * monthly minimum age in Brazil is about US$250.00
